# The interaction between cognitive biases, intolerance of uncertainty and anxiety sensitivity in schizophrenia spectrum disorder

**DOI:** 10.1192/j.eurpsy.2025.2226

**Published:** 2025-08-26

**Authors:** I. Keleş Altun, S. A. Korkmaz, I. Açar Duran, M. I. Atagün

**Affiliations:** 1 Department of Psychiatry, University of Health Sciences Bursa Yüksek İhtisas Training and Research Hospital, Bursa; 2 Department of Psychiatry, Çanakkale Onsekiz Mart University, Çanakkale, Türkiye

## Abstract

**Introduction:**

The cognitive model of psychosis suggests that psychotic symptoms may arise due to biases in information processing. Cognitive biases such as jumping to conclusions (JTC), belief inflexibility (BI), selective attention to threat (AT), and external attribution (EA) are dysfunctional ways of thinking in which distortions are observed in data collection, processing and interpretation. Cognitive biases are known to be associated with the occurrence of positive psychotic symptoms, but evidence for the influence of other cognitive processes on this relationship remains lacking.

**Objectives:**

This study aimed to examine the relationship between cognitive biases and psychotic symptoms in schizophrenia spectrum disorders and the cognitive factors hypothesized to influence this relationship, such as intolerance of uncertainty and insight.

**Methods:**

65 patients with schizophrenia spectrum disorder were included. Sociodemographic data form, Davos Assessment of Cognitive Biases Scale (DACOBS), Intolerance of Uncertainty Scale (IUS) Anxiety Sensitivity Index-3 (ASI-3), and Beck Cognitive Insight Scale (BCIS) Positive and Negative Syndrome Scale (PANSS) and Beck Anxiety Inventory were applied. Ethics committee approval was obtained (no: 2023-40). Statistical analysis was performed with SPSS 25.

**Results:**

The mean age of the participants was 39.27 ± 12.21 years. 63.1% were male (n: 41) and 36.9% (n: 24) were female. Disease duration was 16.95 ± 12.80 years. Hierarchical regression analysis determined that PANNS positive scores were predicted by DACOBS external attribution subscale and IUS, ASI-3 and BCIS scores had a moderator effect (F: 3.51 p<0.001).

**Conclusions:**

Our results showed that external attribution bias is the only cognitive bias associated with positive psychotic symptoms. Intolerance of uncertainty and anxiety sensitivity also play a role in the prediction of positive psychotic symptoms. Targeting intolerance of uncertainty and anxiety sensitivity with cognitive interventions may be useful in the treatment of positive psychotic symptoms in schizophrenia spectrum disorders.

**Disclosure of Interest:**

None Declared

